# Novel Somatostatin Receptor Ligands Therapies for Acromegaly

**DOI:** 10.3389/fendo.2018.00078

**Published:** 2018-03-07

**Authors:** Rosa Maria Paragliola, Roberto Salvatori

**Affiliations:** ^1^Unit of Endocrinology, Università Cattolica del Sacro Cuore, Rome, Italy; ^2^Department of Medicine, Division of Endocrinology, Metabolism and Diabetes, Pituitary Center Johns Hopkins University School of Medicine, Baltimore, MD, United States

**Keywords:** acromegaly, somatostatin receptor ligands, octreotide, lanreotide, pasireotide, somatoprim

## Abstract

Surgery is considered the treatment of choice in acromegaly, but patients with persistent disease after surgery or in whom surgery cannot be considered require medical therapy. Somatostatin receptor ligands (SRLs) octreotide (OCT), lanreotide, and the more recently approved pasireotide, characterized by a broader receptor ligand binding profile, are considered the mainstay in the medical management of acromegaly. However, in the attempt to offer a more efficacious and better tolerated medical approach, recent research has been aimed to override some limitations related to the use of currently approved drugs and novel SRLs therapies, with potential attractive features, have been proposed. These include both new formulation of older molecules and new molecules. Novel OCT formulations are aimed in particular to improve patients’ compliance and to reduce injection discomfort. They include an investigational ready-to-use subcutaneous depot OCT formulation (CAM2029), delivered via prefilled syringes and oral OCT that uses a “transient permeability enhancer” technology, which allows for OCT oral absorption. Another new delivery system is a long-lasting OCT implant (VP-003), which provide stable doses of OCT throughout a period of several months. Finally, a new SRL DG3173 (somatoprim) seems to be more selective for GH secretion, suggesting possible advantages in the presence of hyperglycemia or diabetes. How much these innovations will actually be beneficial to acromegaly patients in real clinical practice remains to be seen.

## Introduction

Trans-sphenoidal surgery is the treatment of choice in acromegaly, because it can provide prompt reduction of GH and IGF-1 levels, thereby improving morbidity and mortality (1). Considering biochemical remission criteria as normal IGF-1 levels and either GH levels < 0.4 ng/mL after oral glucose load or random GH < 1.0 ng/mL (2), successful surgery is obtained in experienced hands in about 80–90% of microadenomas and up to 66% of macroadenomas (3).

Determinants of mortality in acromegaly include a serum GH > 2.5 ng/mL and an elevated IGF-1 as well as hypertension, cardiovascular and cerebrovascular disease, and hypoadrenalism (4–6). Consequently, patients with persistent disease after surgery or in whom surgery cannot be considered, require medical therapy. Somatostatin (SST) receptor (SSTR) ligands (SRLs) octreotide (OCT), lanreotide (LAN), and the more recently approved pasireotide (PAS), a new ligand with broader receptor binding profile, are considered the mainstay in the medical management. The response rate to medical treatment varies in different studies, but the biochemical control rate does not exceed 50–55% of patients (7).

In the attempt to offer a more efficacious and better tolerated medical approach, research has focused to override some limitations related to the use of approved SRLs (including limited efficacy, need for life-long intramuscular or deep subcutaneous injections, and some adverse effects).

## Mechanisms of Action of SRLs

Human SST was isolated in 1973 and identified as the hypothalamic responsible for inhibition of GH secretion (8). However, SST is expressed in several other tissues (central nervous system, endocrine system, and broadly in the gastrointestinal system). SST derives from processing of the 116-aminoacid precursor prepro-SST (8). Enzymatic degradation produces two bioactive proteins: a 14-aminoacid molecule called “SST-14” and a larger 28-aminoacid form “SST-28” (9).

The mechanism by which SST acts on the target cells is complex and not entirely understood. Its physiological effects include the inhibition of GH secretion from both normal pituitary and GH-secreting tumors (8, 10), as well as the inhibition of cell proliferation and the regulation of endocrine and exocrine cells of the digestive tract (11). The potent and broad anti-secretory and anti-proliferative activities of SST represent suitable properties to apply in clinical practice, with the downside of possible broad unwanted effects. However, both native SST isoforms have a short half-life (about 2 min) due to the presence of multiple enzymatic cleavage sites which result in rapid degradation. This limitation has been partially surpassed by the development of more stable and potent analogs. Synthetic SRLs with both a longer half-life and an increased affinity for SSTRs have been developed. For example, the compound “SMS 201–995” (OCT) exhibits a 19-fold and a 3-fold higher potency than native SST on the inhibition of GH and insulin secretion, respectively (12). A d-Phe at the N-terminal and l-Thr at the C-terminal end and the position 8 substitution of l-Trp by d-Trp increase this peptide’s resistance to enzymatic degradation (13). Compared to native SST half-life of 2–3 min, OCT has a half-life of 90–120 min. Furthermore, its pharmacodynamic action lasts up to 8–12 h (14). Another synthetic compound (“BIM 23104,” LAN) was later developed with similar characteristics (15).

The effects of all SRLs are mediated by their interaction with specific G-protein coupled membrane receptors SSTRs. Five isoforms of SSTRs, belonging to the family of, have been described (SSTR 1–5). Each receptor isoform is encoded by genes localized on different chromosomes (15). The gene encoding SSTR2 creates two splice variants, a long (SSTR2A) and a short (SSTR2B) form (16), differing in length and amino acid sequence in their intracellular carboxyl termini. The two isoforms have similar affinity for a number of SSTR2-selective agonists, but they differ in their ability to couple to adenylyl cyclase (17). In pituitary tumors, SSTR2 mRNA is expressed as the 2A variant (18).

The SSRTs’ extracellular domain contains the ligand binding sites, while the intracellular domain is responsible for second messenger activation (19). The affinity of SST-14 and SST-28 is similar for all the receptor subtypes, except for SSTR5 which shows a 10-fold higher affinity for SST-28, suggesting a possible different role for this receptor (20). All five SSTRs are present in the central nervous system, gastrointestinal tract, endocrine and exocrine glands, and inflammatory and immune cells.

The current understanding of SST/SSTRs intracellular signaling is mostly based on *in vitro* models. The interaction between SST and its receptors activates a number of intracellular cascades. It causes decrease in intracellular cAMP (due to inhibition of adenylyl cyclase activity) and a reduction in calcium ion influx due to the activation of potassium and calcium channels. Furthermore, SSTRs activation stimulates both mitogen-activated protein (MAP) kinase and protein phosphatases such as calcineurin (which inhibits exocytosis), phosphotyrosine phosphatases (PTP) (which dephosphorylate growth factor receptor kinases thereby inhibiting mitogenic signaling and cell proliferation), and serine threonine phosphatases (which activate calcium and potassium channel proteins) (21). SSTRs also regulate the phospholipase C and A2 and cyclic guanosine monophosphate, proteins involved in signal transduction (22). Some differences can be found in the intracellular signaling pathways of each SSTRs isoform: all SSTRs are coupled to inhibitory G protein, blocking adenylate cyclase. SSTR1, SSTR2, and SSTR3 transduce their anti-proliferative effect by stimulating one or more PTPs, which in turn affect MAP kinase activity and the survival PI3K pathways. Conversely, SSTR5 mediates its anti-proliferative effect through PTP-independent pathways (23).

Most tumors arising from tissues that express SSTRs maintain this expression, offering the possibility of a therapeutic approach with SRLs. They are gastrointestinal and bronchopulmonary neuroendocrine tumors, as well as several kinds of pituitary tumors, medulloblastoma, and medullary thyroid carcinoma (24). The final anti-proliferative effect occurs by both “direct” and “indirect” mechanisms. The direct effects include promoting both cytostatic signaling and cytotoxic action (by induction of apoptosis). The “indirect” effects act on the tumor microenvironment and include blockade of neo-angiogenesis, inhibition of tumor-promoting signals secretion from immune cells and blockade of paracrine growth factor secretion (23).

## SRLs in the Treatment of Acromegaly

The name “somatostatin” originates from the first appreciated function of this molecule as inhibitor of GH (“somatotropin”) release (25). It is logical that the first use of SRLs focused on their effect in acromegaly, although they are also used for the treatment of TSH and ACTH-secreting adenomas and neuroendocrine tumors in other areas of the body (25).

The most commonly used formulations of SRLs are OCT [subcutaneous and long-acting repeatable/release (LAR)] and LAN [slow release (SR) or aqueous gel formulation autogel (ATG)] (“first-generation SRLs”). Recently, approval for subcutaneous PAS for Cushing disease (CD) and (26) PAS-LAR for acromegaly was granted both in Europe and the United States.

First-generation SRLs are often considered the first-line medical treatment in acromegaly. The safety profile of these SRLs is good. The side effects are mainly the result of the activation of the receptors expressed in the gastrointestinal tract (cholelithiasis, effects on glucose metabolism and gut motility), as well as injection site side effects (27). The response to SRLs (control of hormonal hypersecretion and tumor shrinkage) in acromegaly is variable. The biochemical response varies in different studies, ranging between 20 and 80% (28, 29). The different degree of response can be due to clinical parameters and to histopathology and molecular mechanisms, including SSTR expression or activity (30). Most GH-secreting pituitary tumors (more than 95%) express SSTR2, followed by SSTR5 (more than 85%) and SSTR3 and SSTR1 (both in more than 40%). SSTR4 expression is rare (25).

The predominant expression of SSTR2 and SSTR5 represents the basis for the clinical use of OCT and LAN, molecules that have high affinity for these receptors (SSTR2 > than SSTR5) (31). Because SSTR2 and SSTR5 regulate the release of GH from somatotropic cells, the primary pharmacodynamic effect of both OCT and LAN is a reduction of GH and/or IGF-1 serum levels (32). In addition, SSTR2 and SSTR5 can result in tumor shrinkage by inhibiting cell proliferation and/or activating cell apoptosis (33).

The main factor causing tumor resistance to OCT and LAN is the absent or reduced SSTR density (34). Indeed, GH suppression and tumor shrinkage induced by SRLs correlate with SSTR2 mRNA levels, and OCT resistance in GH-secreting adenomas occurs due to loss of SSTR2A expression (35). Beta-arrestins are proteins that bind G-protein-coupled receptors and block further signaling by preventing receptor re-circulation to the membrane. These proteins have been recently considered as important regulator on SSTR2 function (36). Low beta-arrestin expression and high SSTR2/beta-arrestin ratio have been reported to be associated with SRLs responsiveness (36). However, these results have been questioned by a recent study (37).

Clinically relevant factors predicting poor SRLs efficacy include young age, large tumor size, high basal levels of GH, and “sparsely granulated” adenoma at histology (which correlates with a specific radiological pattern at magnetic resonance imaging (MRI) and is associated with a lower SSTRA2 expression than “densely granulated” tumor) (30, 38). Heterozygous loss-of-function mutations in the aryl hydrocarbon receptor interacting protein (AIP) are associated with young-onset GH secreting pituitary adenomas (39). It has been demonstrated that both familial and sporadic *AIP* mutations are associated with poor response to first-generation SRLs (40) and that reduced AIP expression, even without mutation, is associated with first-generation SRLs resistance (25). This is probably due to the reduced expression of SSTR2A in AIP-deficient tumors (41).

Pasireotide has high affinity to SSTR1, SSTR2, SSTR3, and SSTR5. Compared to OCT, it has a 40-fold higher affinity and a 158-fold higher functional activity for SSTR5 (42). Interestingly, AIP-deficient tumors resulted equally responsive to PAS compared to non-AIP-deficient tumors, without any difference in SSTR5 expression (41). Accordingly, in *AIP* knockout mouse models of pituitary adenoma PAS is effective in controlling IGF-1 levels, while OCT does not show any significant effect (43). Consequently, low AIP expression seems not to be a relevant predictor factor of poor response to PAS.

## First Generation SRLs: OCT and LAN

### Octreotide

Octreotide was the first SRL introduced in clinical practice, and it is still widely used for the treatment of acromegaly. OCT has high-affinity binding to SSTR2 and SSTR5. Cell cultures from SSTR2 receptor-deficient mice suggest that OCT mediates its pharmacological action primarily via the SSRT2 receptor (44). Initially, OCT was a subcutaneous or intravenous preparation, requiring a three daily injection regimen. The observation that continuous infusions were more effective than subcutaneous regimen suggested that preparations causing a sustained level of the drug may be beneficial (45, 46). Consequently, an intramuscular depot preparation using microspheres was introduced (OCT-LAR), allowing for monthly injections and facilitating the use of the drug. The mean time needed for the OCT-LAR 20 and 60 mg doses to reach maximum concentration is 22 and 12.6 days, respectively, with an inter-subject variability in mean maximum drug concentration of 32 and 38% (47). The profile of OCT-LAR is characterized by a transient increase in OCT blood level on day 1, followed by a lag phase (days 2–6) of decreased concentration. After 6–8 days, a new increase occurs, reaching a plateau concentration maintained for about 30 days (47).

The effects of this formulation have been demonstrated to be dose and time dependent (48), and a significant percentage of patients treated with OCT-LAR achieved biochemical control of acromegaly, a percentage much higher than those already reported for regular OCT (49). However, the reported biochemical efficacy varies among different studies. In some clinical trials the percentage of patients reaching biochemical control of acromegaly is up to 70%, while surveys including non-selected cohort of patients report a biochemical control in less than 50% of treated patients (49). A large meta-analysis demonstrated that OCT-LAR induces tumor shrinkage in 66.0% of patients, with a mean percentage reduction in tumor size of 50.6% (50).

### Lanreotide

Lanreotide is an octapeptide analog of natural SST binding with high affinity to SSTR2 and with a minor affinity to SSTR5 (32). Two formulations have been developed: a LAN-SR obtained by combining LAN with microspheres of lactide/glycolide copolymers, allowing administration every 7–28 days (51), and LAN-ATG, which is a viscous aqueous formulation in ready-to-use prefilled syringes that are administered every 28–56 days (52). The mean time needed for the LAN-ATG 90 and 120 mg doses to reach maximum concentration is 2.4 and 1.1 days, respectively, with inter-subject variability of 52 and 84% for 90 and 120 mg doses, respectively. The profile of LAN-ATG is characterized by a peak concentration on day 1, followed by a constant decrease throughout the treatment period (47). First-line therapy with LAN-SR for 6–48 months achieved a significant (20–25%) tumor volume reduction in 22–50% of patients and a good biochemical control (53, 54), while patients treated with LAN-ATG obtained a tumor volume reduction (> 20%) after 48 weeks of treatment with 120 mg in the 63% of cases (55). The percentage of patients reaching biochemical control ranges between 63 and 78% and between 65 and 70% for GH and IGF-1, respectively (53, 54). Despite lack of head-to-head studies, it is generally believed that OCT-LAR and LAN-ATG have similar biochemical efficacy in acromegaly (56). A recent large international clinical trial showed that patients previously biochemically controlled with OCT LAR every 4 weeks are possible candidates for LAN-ATG 120 mg every 6 and 8 weeks, with the advantage of reducing the frequency of injections (57). It has recently been shown that LAN-ATG “high-frequency” (120 mg/21 days) and “high-dose” (180 mg/28 days) regimens were effective in normalizing IGF-1 in one-third of patients who were incompletely controlled by conventional SRLs therapy (58).

## Pasireotide

Pasireotide (SOM 230), a “second generation” SRL, is a multireceptor-targeted SST generated by introducing four synthetic and two essential amino acids of SST in a cyclohexapeptide structure. PAS has high affinity for four of the five human SSRTs: it has a 30-, 11-, and 158-fold higher activity than OCT on SSTR1, SSTR3, and SSTR5, respectively, with a 7-fold lower activity on SSTR2 (59). PAS has a potent effects on GH release (60). A long-acting form has been developed (PAS-LAR) with identical delivery system to OCT-LAR. Like OCT-LAR, it is administered via deep intramuscular injection. The recommended initial dose is 40 mg every 28 days (61), which may be increased to a maximum of 60 mg every 28 days (62).

In a 12-month randomized phase III double-blind, multicenter study including 358 medication-naïve patients, PAS-LAR was shown to be more effective than OCT-LAR in serum IGF-1 and GH normalization. A significant (≥20%) tumor volume reduction was seen in 80.8% of PAS-LAR- and 77.4% of OCT-LAR-treated patients, without difference between the post-surgery and *de novo* groups (63). In the 12-months cross-over extension phase (in which patients crossed to opposite treatment), further tumor volume reduction was seen in a mean of 25% of cases with PAS-LAR and 18% with OCT-LAR. Subjects with inadequate biochemical control at end of the study were eligible to switch to PAS-LAR or OCT-LAR. Twelve months later, 17.3% of PAS-LAR and none of OCT-LAR patients achieved control. The extension phase (64) of the core study, involving 120 patients who continued their randomized therapy, was aimed to evaluate the efficacy and safety of PAS-LAR and OCT-LAR for up to 26 months (64). Biochemical control was maintained for up to 25 months during PAS-LAR treatment. The safety profile of PAS-LAR is comparable to other SRL’s, except for significantly higher frequency and degree of hyperglycemia (seen in 62.9% of PAS-LAR vs. 25.0% of OCT-LAR treated patients) (64).

Another phase III trial (“PAOLA”) enrolled patients previously uncontrolled by first generation SRLs, who were either switched to PAS-LAR or remained on the previous treatment. After 6 months, 15% of PAS-LAR 40 mg and 20% of PAS-LAR 60 mg patients reached biochemical control, while no patients was controlled in the group that remained on first-generation SRLs. More patients receiving 40 mg (18.5%) and 60 mg PAS-LAR (10.8%) had total volume reduction of more than 25% than did those who remained on the ineffective therapies (1.5%) (65).

A recent study (PAPE study) assessed the efficacy and safety of PAS-LAR in 61 acromegaly patients who were well-controlled with first generation of SRLs and weekly pegvisomant. The switch to PAS-LAR, either as monotherapy or in combination with pegvisomant, controlled IGF-1 levels in the majority of patients. Interestingly, in 15 (24.6%) patients, IGF-1 levels remained controlled on PAS-LAR 60 mg alone. Overall, PAS-LAR had a pegvisomant-sparing effect of 66% of the dose compared to the combination of pegvisomant with first generation SRLs. As in other PAS studies, hyperglycemia was the most important safety issue (66).

## Somatoprim

DG3173 (PTR-3173 or somatoprim) is a compound that has been identified while screening SRL’s with a SSTR affinity that would be most selective for suppression of GH secretion (67). This compound binds to human SSTR 2, 4, and 5. *In vitro* inhibitory effects of somatoprim in human fetal pituitary glands and in GH–secreting adenomas on GH secretion are similar to those of OCT (68). A study in GH-secreting adenoma cultures showed effectiveness in reduction in GH secretion by somatoprim in 10 of 21 tumors, broader when compared with OCT (5 of 21) (69). The most attractive feature of this drug is the apparent absence of inhibitory effect on insulin secretion. In fact, studies performed in rats found that somatoprim is 1,000-and 10,000-fold more potent in inhibiting GH release than glucagon and insulin release, respectively (67). The mechanism that mediates this “protective effect” on pancreas is unclear (7). These data suggest that this molecule may turn out to be a suitable option for patients who are unable to tolerate the hyperglycemic or diabetogenic effects commonly associated with SRLs and in particular with the use of PAS (67). Adding an additional attractive feature, a study performed on the non-obese diabetic murine model of insulin-dependent diabetes showed that this drug has a positive effect on renal/glomerular hypertrophy, albuminuria, and changes in glomerular filtration rate (70).

A single-dose, randomized crossover study of healthy volunteers treated with OCT, somatoprim or placebo, confirmed that somatoprim had much less effects on insulin and glucagon release as well as on glucose control compared to OCT (71). An interesting finding is that a positive response to somatoprim is more likely in “sparsely granulated” tumors than in “densely granulated” tumors (69), contrary to what observed for first generation SRLs (but not for PAS) (41). Somatoprim has a pharmacokinetic profile similar to that of OCT (7). Somatoprim is not yet available commercially.

## Novel OCT Formulations

### CAM2029

The presently available OCT-LAR has been obtained by combining OCT with microspheres of carboxymethylcellulose that allows to increase its therapeutic action to 24–42 days (27). These microspheres consist of a biodegradable glucose star polymer that degrades mostly through hydrolysis. However, drug release is not linear and OCT-LAR injections need to be administered intramuscularly, with a rather large needle (19 G). Therefore, alternative long-acting delivery systems that avoid variations in drug absorption and allow for smaller needle would be of benefit. CAM2029 is an investigational ready-to-use subcutaneous depot OCT formulation, delivered via prefilled syringes, aimed to address the limitation of LAR (7). This formulation is a liquid solution based on naturally occurring lipids, which can be administered by thin needles (22–27 G). The drug is injected into subcutaneous or intramuscular tissue. The depot formulation absorbs interstitial aqueous fluid, resulting in a highly viscous liquid-crystal gel phase (72), a spontaneous process deriving from lipid self-assembly. The instantaneous gel formation determinates an effective encapsulation of the drug from the depot matrix, which assures a fast initial release (without the initial peak observed with OCT-LAR) followed by a consistent slow drug release. The depot is eventually biodegraded in the subcutaneous or intramuscular tissue (73). Additionally, while OCT-LAR requires refrigeration and reconstitution before injection, CAM2029 remains stable at room temperature (73), which may allow self- or partner home administration.

A phase I randomized open label study aimed to assess the pharmacokinetics, pharmacodynamics, safety, and tolerability of subcutaneous CAM 2029 has been performed in healthy volunteers, showing that this preparation had approximately fourfold to fivefold greater OCT bioavailability, with more rapid onset and similar duration of effect in terms of suppression of IGF-1, when compared with OCT-LAR (72). CAM2029 administration was well tolerated, both locally and systemically. As expected, the most frequent adverse events for both CAM 2029 and OCT-LAR were mild-to-moderate gastrointestinal events. CAM2029 showed a rapid onset and sustained release for up to 4 weeks, with a more stable drug release compared to OCT-LAR formulation (72). CAM2029 can be administered a frequency not yet determined but likely to be every 4 weeks (7). CAM2029 is not yet available commercially.

### Oral OCT

Acromegaly represents a chronic condition, and the need for injectable drugs has negative effects on patients’ quality of life (74). Therefore, the possibility of an oral administration of SRLs has been evaluated since the first introduction of OCT in clinical practice. However, orally ingested OCT failed to achieve therapeutic drug levels following absorption in the jejunum, due to the intestinal barrier and too low and very variable enteral absorption were reported (75, 76).

A new interest for the oral administration grew after the introduction of new technologies, the so called “transient permeability enhancer (TPE)” which facilitates the intestinal absorption of molecules by transient opening of intestinal epithelial tight junctions. With this technique, intestinal permeability is related to molecular size: as demonstrated *in vivo* in rats with dextran molecules, the highest absorption occurred for the smallest dextran molecules (4 kDa). This feature represents a crucial point, because it reduces the risk of internalization of larger intestinal pathogens or immunoglobulins (7, 77). TPE technologies have been used to enhance OCT oral absorption (78, 79). These OCT capsules consist of a TPE in a medium-chain fatty acid salt sodium caprylate and inert excipients, combined with OCT in an oily suspension and encapsulated in an enteric coating (77). The enteric coating prevents the breakdown before reaching the small intestine. Due to transient opening of tight junctions, OCT can traverse the open junctions resulting in improved absorption. The permeation effect caused by TPE lasts only for 1–2 h (77). *In vivo* primates’ studies showed comparable OCT drug levels between oral OCT capsule and subcutaneous injections. In particular, rapid reduction of GH levels after ingestion of the capsule occurred, and GH remained undetectable for more than 2 h. Safety assessment of after 9 months of daily oral OCT in monkeys showed no systemic toxic effects or organ damage, and no differences in toxicity compared to the injectable OCT (77). In particular, no signs of inflammation were documented with either formulation on gastrointestinal epithelia and mucosa.

In a phase I study conducted in 75 healthy volunteers, oral doses of 3, 10, or 20 mg of OCT and a single subcutaneous injection of 0.1 mg OCT were administered (78). Because of the low baseline GH secretion (80), the effect on GH was tested also using the GHRH/arginine stimulation test (81). Both basal and GHRH/arginine-stimulated GH levels were significantly suppressed by a single oral OCT dose (78). Oral OCT absorption resulted in a dose-dependent increase in systemic OCT exposure. Twenty milligrams of orally administrated OCT resulted in exposure similar to that of subcutaneous injection of 0.1 mg. Both oral and subcutaneous OCT treatments were well tolerated.

After this study, oral OCT was studied in a phase III multicenter, open-label, dose-titration, baseline-controlled study in acromegaly patients (82). One hundred and fifty-five patients receiving injectable SRLs with complete or partially control were switched to 40 mg/day oral OCT capsules. The drug was administered in two divided doses (morning and evening) ≥1 h before and ≥2 h after meals. The dose was increased to 60 and then up to 80 mg/day if needed, depending on serum IGF-1 levels. Subsequently, fixed doses were maintained for a 7-month treatment and a voluntary 6-month extension. One hundred and fifty-one patients underwent at least one biochemical assessment after the first oral dose. Of these, 65% maintained biochemical response at the end of the treatment period (7 months) and 62% at the end of extension treatment (up to 13 months). Predictors of responsiveness to oral OCT included good previous control and low to mild doses of injectable SRLs. About 89% of subjects experienced an adverse effect, including gastrointestinal, neurological, and musculoskeletal side effects, consistent with the known profile of this drug, but clinical control of symptoms related to acromegaly improved during the trial (82). Gastrointestinal infections were not increased and only a single case of viral gastroenteritis was observed (82).

As mentioned before, the response to OCT therapy is in general related to SSTR status of the tumor and the biochemical control is dependent by this status as well as by disease activity and by and drug levels. However, it has not yet well established if these factors are different for oral or injectable OCT (83). Oral OCT is not yet available commercially.

### OCT Subcutaneous Implants

Another possible approach to SRL delivery is based in long-lasting subcutaneous implants. OCT implant (VP-003) hydrogel formulation, which provides stable doses of OCT throughout a period of 6 months, has been evaluated in two phase II open-label randomized studies in patients with acromegaly with previously demonstrated responsiveness to OCT (84). Implants were inserted after local anesthesia subcutaneously in the inner aspect of the upper arm. In one study, one or two 52 mg implants were placed (“52 mg study”). Five patients received one implant and six received two. In another study (“84 mg study”), 17 patients received a hydrated 17 a non-hydrated 84 mg implant. Implants were removed after 6 months. In the 52 mg study (which included a wash-out period), GH levels declined from baseline values during the first month and remained significantly suppressed during the 6-month treatment: 3 of 11 patients (27%) in the 52 mg study and 17 of 33 patients (52%) in the 84 mg study achieved IGF-1 normalization. GH levels were < 2.5 ng/mL in 73 and 39% of patients of 52 and 84 mg study, respectively. Despite higher OCT area under the curve with hydrated implants (mostly due to higher release during the initial 6 weeks), no significant difference in efficacy between the two preparations was observed. The side effects were mostly limited to the gastrointestinal system and were generally mild to moderate in severity (84).

A phase III open label study performed in 163 subjects was aimed to validate that an OCT implant (84 mg) is safe and efficacious in acromegaly patients responsive to prior monthly OCT-LAR injections. The study confirmed that OCT implant maintained normal blood levels of GH and IGF-1 (86 vs. 84% for OCT-LAR) for 6 months. Diarrhea and headache were more frequent with the implant, whereas cholecystitis and hypertension were more frequent with OCT-LAR (85). This formulation is not yet available commercially.

## Conclusion and Future Perspectives

The main goals of the medical treatment of acromegaly patients are represented by control of tumor growth and normalization of GH and IGF-1 hypersecretion, as well as by a clinical control of acromegaly related symptoms. SRLs have represented a cornerstone of medical treatment of acromegaly for about 40 years. However, even if extraordinary progresses have been obtained in this field, the achievement of disease control with currently approved SRLs is sometimes precluded by incomplete efficacy, adverse effects, and need for parenteral administration. Recent research strategies were aimed to improve the efficacy, tolerability, and compliance. These include new molecules and new formulation of older molecules. New formulations could offer easier or less frequent routes of administration. New molecules cold offer increased efficacy in a broader percentage of patients. Despite the understandable enthusiasm for advances in the therapeutic armamentarium, it is important to remain critical about the theoretical superiority of a newly developed drug (86). Indeed, how much these innovations will actually be beneficial to acromegaly patients in real life remains to be seen. Furthermore, due to the complex processes and different mechanisms involved in pathogenesis of acromegaly, it seems unlikely that a single agent will be considered the “perfect agent” for all patients. Investigation into the biological heterogeneity of GH–secreting tumors, predicting response to therapy according to individualized assessments of different SSTRs’ expression, tumor size, invasiveness, MRI appearance, granularity, and gene expression, could offer new “personalized” targets for medical treatment.

## Author Contributions

The authors contributed equally to this work.

## Conflict of Interest Statement

RS participates to multi-center research projects sponsored by Pfizer and Novartis, and has received patient education support grants from Ipsen. He has received consulting fees from Pfizer.

## Abbreviations

ATG, autogel; CD, Cushing disease; GH, growth hormone; IGF-1, insulin like growth factor 1; LAN, lanreotide; MAP, mitogen-activated protein; MRI, magnetic resonance imaging; OCT, octreotide; PAS, pasireotide; PTP, phosphotyrosine phosphatases; SR, slow release; SRLs, somatostatin receptor ligand; SST, somatostatin; SSTR, somatostatin receptor; TPE, transient permeability enhancer.
